# Supplementary material for: Bacterial microcompartments and energy metabolism drive gut colonization by Bilophila wadsworthia
Source: Nat Commun. 2025 May 30;16:5049. doi: 10.1038/s41467-025-60180-y (PMC12125255; doi:10.1038/s41467-025-60180-y)
Supplement: Supplementary file 1 — Supplementary Information [file 41467_2025_60180_MOESM1_ESM.pdf]

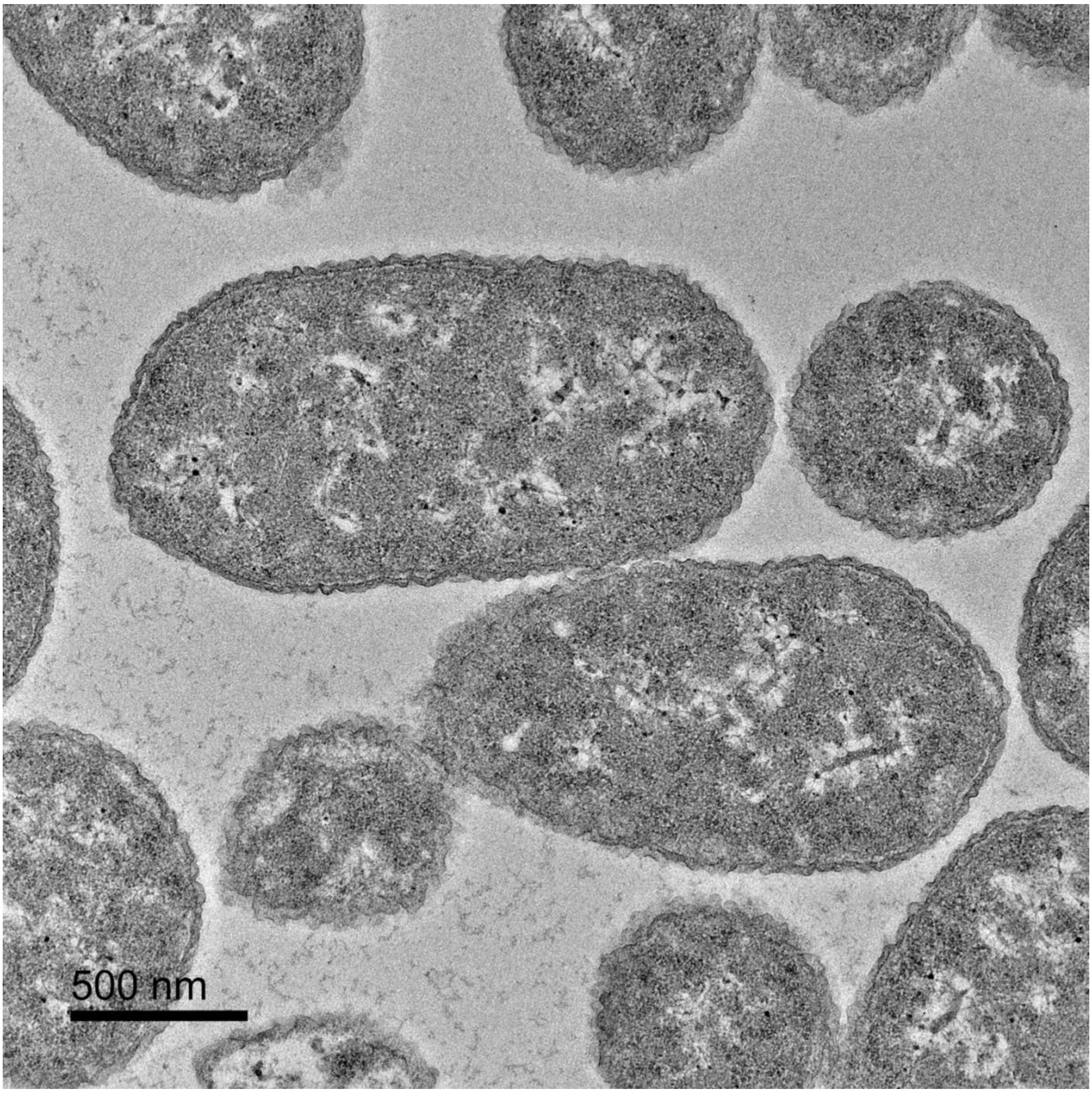

Supplementary Figure 1. Transmission electron microscopy of *B. wadsworthia in vitro.* The cultures were grown overnight in Postgate C media supplemented with 10 mM taurine. Microcompartments were not observed in two independent cultures of *B. wadsworthia* grown in Postgate C medium or in one culture grown in anaerobe basal broth (ABB) supplemented with 10 mM taurine.

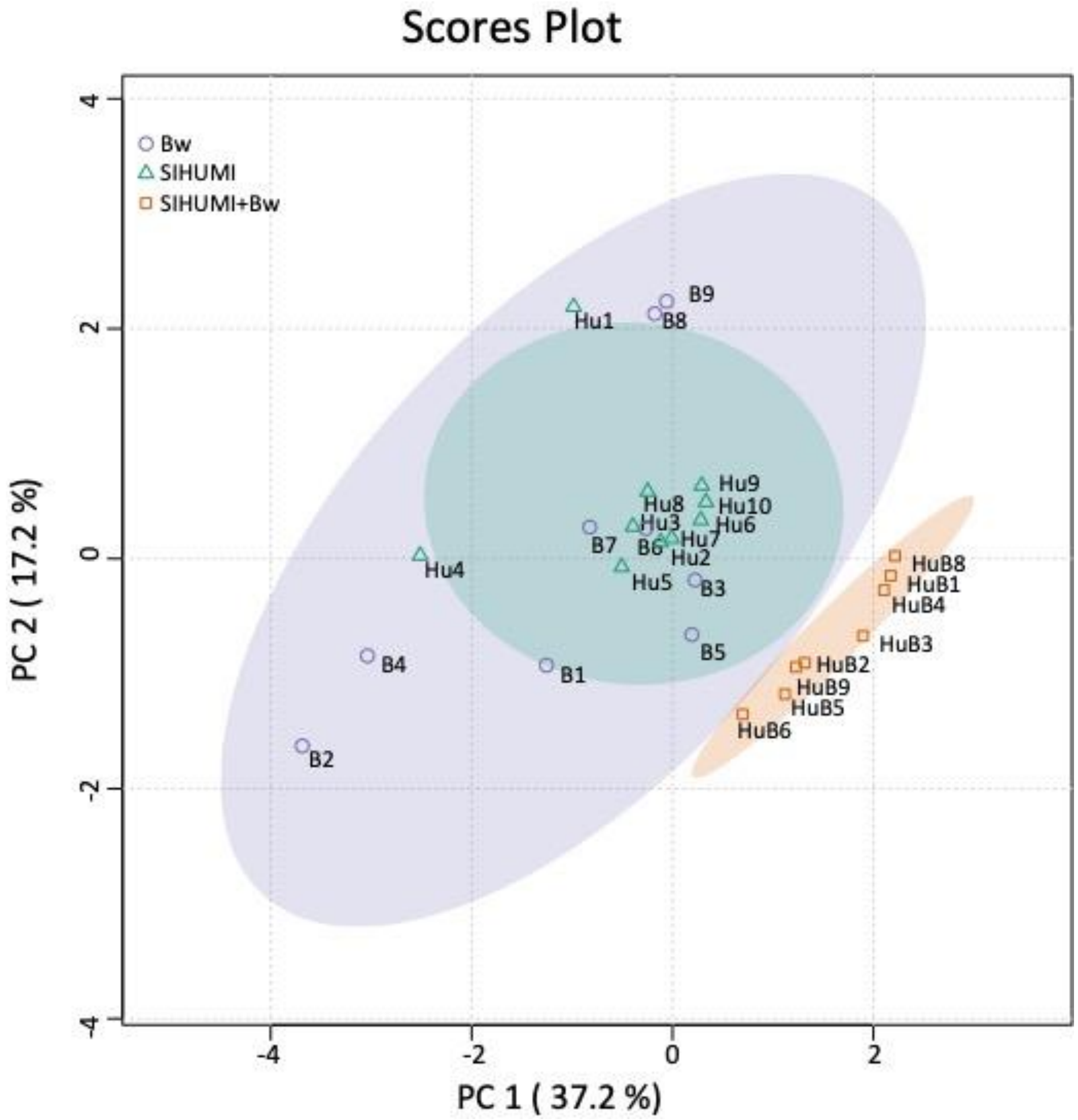

Supplementary Figure 2. Score plot of the metabolome quantified using H^1-NMR from faecal water samples. Each point on the plot represents a biological replicate collected from the same time point (refer to Figure 1b for context on experimental design). The score plot was generated using MetaboAnalyst v.5.0 based on Principal Component Analysis (PCA), showing the variance between biological replicates in terms of their overall metabolite profiles. The clustering of points reflects the metabolic similarity between replicates. Statistical significance was assessed using PERMANOVA (Permutational Multivariate Analysis of Variance) with 999 permutations. F-value: 9.7383; R-squared: 0.44798; and p-value: 0.001, indicating significant differences between the groups. Data represent biological replicates derived from individual mice: SIHUMI (N=10), Bw (N=9), and SIHUMI+Bw (N=8).

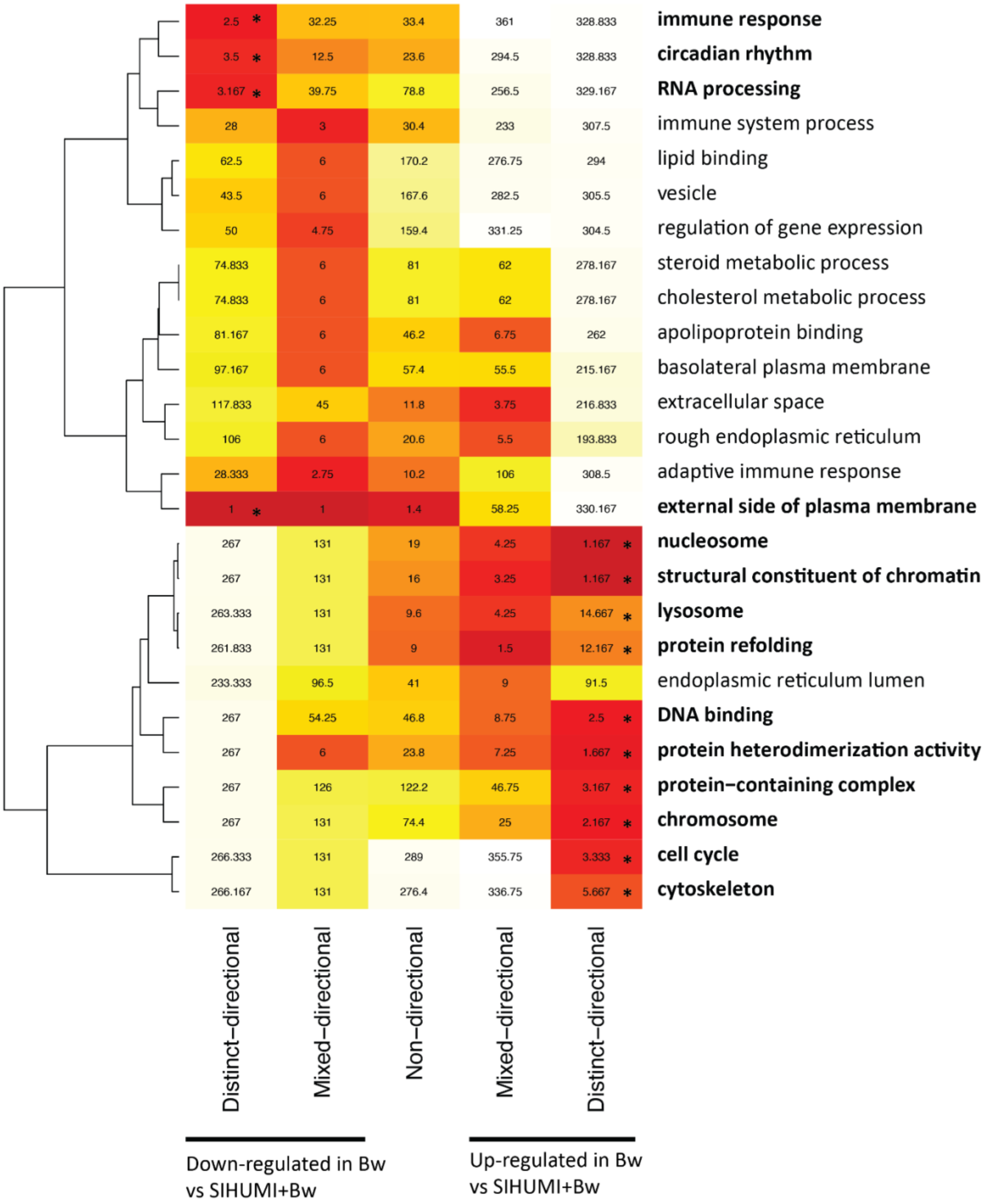

Supplementary Figure 3. Heatmap of consensus scoring of the gene set enrichment analysis (GSEA) of the mouse host. Gene sets that received a median rank <10 are included by Piano (incorporated into the RNAflow pipeline). *p-value <0.01. Differential gene expression was calculated from Bw (N=6), and SIHUMI+Bw (N=6).

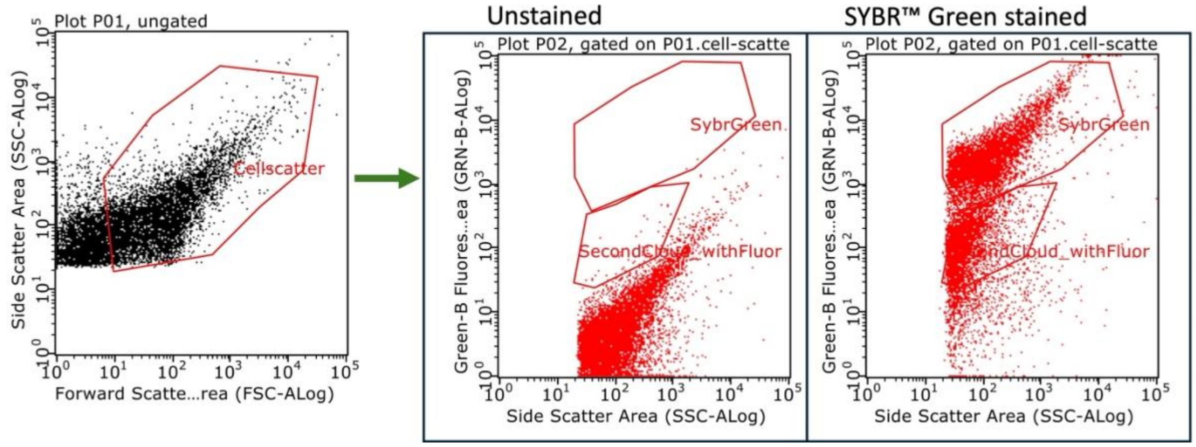

Supplementary Figure 4. Gating strategy used to determine the number of bacterial cells in faecal stool samples from mice. The gating sequence involves initial selection based on cell scatter properties (left panel), followed by identification of bacterial cells using Sybr^{TM}Green staining using as control the unstained sample. All stool samples had a non-stained control. The example shown is from a mouse gavaged with the SIHUMI consortia.

a)

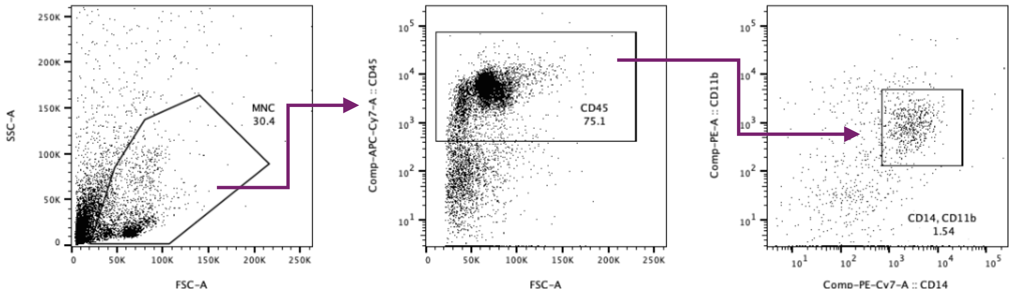

b)

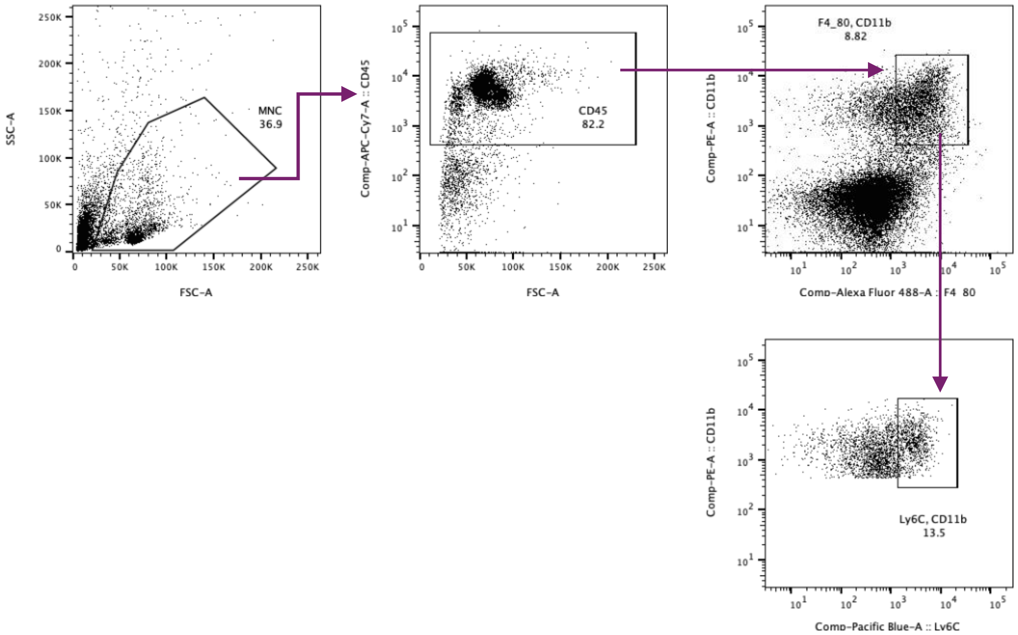

Supplementary Figure 5. Gating strategy used to determine the macrophages from liver tissue. a) Gating strategy for CD11b+/CD14+ cells isolated from fresh liver tissues. **b)** Gating strategy for CD11b^{+}/F4-80^{+} and CD11b^{+}/F4-80^{+}/Ly6C^{+} cells isolated from fresh liver tissues.
