# Supplementary material for: Bacterial microcompartments and energy metabolism drive gut colonization by Bilophila wadsworthia
Source: Nat Commun. 2025 May 30;16:5049. doi: 10.1038/s41467-025-60180-y (PMC12125255; doi:10.1038/s41467-025-60180-y)
Supplement: Supplementary file 2 — Description of Additional Supplementary Files [file 41467_2025_60180_MOESM2_ESM.docx]

**File Name: Supplementary Data 1**

**Description:** Non-essential genes *in vivo* showing a significant logFC compared to *in vitro* conditions in at least one condition were determined using AlbaTraDIS. Negative values represent genes that had lower mutation frequencies in the *in vivo* conditions and are therefore important for colonization. For transcriptomes, positive logFC indicates higher expression *in vivo* compared to *in vitro*. Genes were classified using BV-BRC. Analyses were conducted for all animals, with the following group sizes: SIHUMI+Bw (N=6), SIHUMI (N=10), and Bw (N=9) for organs; and SIHUMI+Bw (N=8), SIHUMI (N=10), and Bw (N=9) for stool. Sequencing reads from multiple animals were concatenated into two random groups per tissue material to increase sequencing depth.

**File Name: Supplementary Data 2**

**Description:** Protein comparison of *B. wadsworthia* and other representative strains was conducted to identify conserved and unique proteins using as reference the *B. wadsworthia* QI0013 genome.

**File Name: Supplementary Data 3**

**Description:** Transcriptome read counts of *Bilophila wadsworthia* *in vitro* and in caecal material. Only reads with a minimum count of 3 and a minimum count per million (CPM) of 3 in at least 3 samples are included. Differential expression analysis was performed using the Voom/Limma method. P-values were adjusted for multiple comparisons using the Benjamini–Hochberg method to control the false discovery rate. The analysis is based on RNA-seq data from SIHUMI+Bw (N=6) and Bw (N=6) groups. Transcriptomic datasets can be interactively visualized under the Degust platform at this link (https://degust.erc.monash.edu/degust/compare.html?code=b204766988adfcd5afd7d6f12d71d87e#/) for an in-depth comparison of gene expression data. The animals were randomly selected for RNA-seq.

**File Name: Supplementary Data 4**

**Description:** Metabolite concentrations determined using H1NMR (mM.kg-1). Data were transformed using square root transformation and range scaling (mean-centered and divided by the range of each variable) via MetaboAnalyst for statistical analysis. P-values were estimated using a linear model with fixed effects for group and random effects for cage. FDR-adjusted p-values from p.adjust, considering all pairwise comparisons together, are shown; however, they were only considered for metabolites that were not specifically targeted based on the TraDIS and metatranscriptomic analyses. Groups: SIHUMI+Bw (N=8), SIHUMI (N=10), Bw (N=9).

**File Name: Supplementary Data 5**

**Description:** Transcriptome read counts from caecal material using as reference genomes the SIHUMI consortia and *B. wadsworthia*. Reads were normalized to counts per million (CPM). The analysis is based on RNA-seq data from SIHUMI+Bw (N=6) and Bw (N=6) groups. The animals were randomly selected.

**File Name: Supplementary Data 6**

**Description:** Differentially expressed mouse host genes were detected using DESeq2, which applies a two-sided Wald test. P-values were adjusted for multiple comparisons using the Benjamini–Hochberg method to control the false discovery rate. Only genes with adjusted P < 0.05 are included. Genes with log2FC values > 0 are more highly expressed in the *B. wadsworthia* group, while those with log2FC values < 0 are more highly expressed in the SIHUMI+Bw group. Genes involved in the alcoholism pathway (mmu05034), which was found to be enriched using WebGestalt, are shown in the Enrichment Pathway section. This analysis is based on RNA-seq data from SIHUMI+Bw (N=6) and Bw (N=6) groups, with animals randomly selected.

**File Name: Supplementary Data 7**

**Description:** Primers used for TraDIS sequencing
