# Supplementary material for: Bacterial microcompartments and energy metabolism drive gut colonization by Bilophila wadsworthia
Source: Nat Commun. 2025 May 30;16:5049. doi: 10.1038/s41467-025-60180-y (PMC12125255; doi:10.1038/s41467-025-60180-y)
Supplement: Supplementary file 4 — Reporting Summary [file 41467_2025_60180_MOESM4_ESM.pdf]

nature portfolio

Corresponding author(s): Dr Lizbeth Sayavedra

Last updated by author(s): May 5, 2025

Reporting Summary

Nature Portfolio wishes to improve the reproducibility of the work that we publish. This form provides structure for consistency and transparency in reporting. For further information on Nature Portfolio policies, see our Editorial Policies and the Editorial Policy Checklist.

Statistics

For all statistical analyses, confirm that the following items are present in the figure legend, table legend, main text, or Methods section.

n/a	Confirmed	
☐	☒	The exact sample size (*n*) for each experimental group/condition, given as a discrete number and unit of measurement
☐	☒	A statement on whether measurements were taken from distinct samples or whether the same sample was measured repeatedly
☐	☒	The statistical test(s) used AND whether they are one- or two-sided<br>*Only common tests should be described solely by name; describe more complex techniques in the Methods section.*
☐	☒	A description of all covariates tested
☐	☒	A description of any assumptions or corrections, such as tests of normality and adjustment for multiple comparisons
☐	☒	A full description of the statistical parameters including central tendency (e.g. means) or other basic estimates (e.g. regression coefficient) AND variation (e.g. standard deviation) or associated estimates of uncertainty (e.g. confidence intervals)
☐	☒	For null hypothesis testing, the test statistic (e.g. *F*, *t*, *r*) with confidence intervals, effect sizes, degrees of freedom and *P* value noted<br>*Give P values as exact values whenever suitable.*
☒	☐	For Bayesian analysis, information on the choice of priors and Markov chain Monte Carlo settings
☒	☐	For hierarchical and complex designs, identification of the appropriate level for tests and full reporting of outcomes
☒	☐	Estimates of effect sizes (e.g. Cohen's *d*, Pearson's *r*), indicating how they were calculated

Our web collection on statistics for biologists contains articles on many of the points above.

Software and code

Policy information about availability of computer code

Data collection	FlowJo software (v.10.8.1), InCyte (v.3.4)
Data analysis	Pathway Tools (v.26); MetaboAnalyst v.6.0; InCyte (v.3.4); bbduk (v.38.06); Unicycler (v.0.4.8); Pilon (v.1.23); CheckM (v.1); BV-BRC; METABOLIC (v.4); RaxML (v.8.2.12); fastANI (v.1.3.4); BioTraDIS; AlbaTraDIS. Custom code to create some of the graphs is available at GitHub: github.com/lsayaved/Bilophila archived on Zenodo as: 10.5281/zenodo.15356479

For manuscripts utilizing custom algorithms or software that are central to the research but not yet described in published literature, software must be made available to editors and reviewers. We strongly encourage code deposition in a community repository (e.g. GitHub). See the Nature Portfolio guidelines for submitting code & software for further information.

Data

Policy information about availability of data

All manuscripts must include a data availability statement. This statement should provide the following information, where applicable:

- Accession codes, unique identifiers, or web links for publicly available datasets
- A description of any restrictions on data availability
- For clinical datasets or third party data, please ensure that the statement adheres to our policy

TraDIS sequencing data was submitted under the project PRJNA1115966. Transcriptomic sequencing data of the caecum is available under project PRJNA1113627. The genome of Bilophila wadsworthia QI0013 was submitted under project PRJNA1085689. The plasmid pQtnpATn5CmPv2.1 is available from Keith Turner.

Research involving human participants, their data, or biological material

Policy information about studies with human participants or human data. See also policy information about sex, gender (identity/presentation), and sexual orientation and race, ethnicity and racism.

Reporting on sex and gender	We isolated Bilophila wadsworthia from a single human stool sample. The sex and gender of the donor are anonymous.
Reporting on race, ethnicity, or other socially relevant groupings	N/A
Population characteristics	N/A
Recruitment	Recruitment for the QIB colon model is registered at ClinicalTrials.gov identifier (NCT number): NCT02653001. The inclusion criteria was the following:<br>Live or work within 10 miles of the Norwich Research Park.<br>Having a normal bowel habit; assessed as regular defecation between 3 times a day and 3 times a week, with a form similar to 3-5 on the Bristol Stool Chart.<br>Do not have any diagnosed chronic gastrointestinal health problem, such as irritable bowel syndrome, inflammatory bowel disease, or coeliac disease.<br>Aged between 25 and 54 years.
Ethics oversight	Human stool collection was approved by the Quadram Institute Bioscience Human Research Governance Committee (IFR01/2015) and by the London-Westminster Research Ethics Committee (15/LO/2169). The trial was registered at clinicaltrials.gov (NCT02653001). The participant provided signed informed consent prior to donating samples. The study was conducted in accordance with the Declaration of Helsinki.

Note that full information on the approval of the study protocol must also be provided in the manuscript.

Field-specific reporting

Please select the one below that is the best fit for your research. If you are not sure, read the appropriate sections before making your selection.

- [x] Life sciences
- [ ] Behavioural & social sciences
- [ ] Ecological, evolutionary & environmental sciences

For a reference copy of the document with all sections, see nature.com/documents/nr-reporting-summary-flat.pdf

Life sciences study design

All studies must disclose on these points even when the disclosure is negative.

Sample size	HF; N=8-10 mice per group. The sample size for the experiment was determined prior to its initiation. In the absence of preliminary data, we selected a target of 10 mice per group, with 5 mice per cage, based on sample sizes used in previous studies investigating the effects of Bilophila wadsworthia on host health (e.g., Natividad et al.2018). No a priori power calculation was performed due to the lack of available preliminary data. Males were selected to minimize variability associated with the hormonal fluctuations present in female mice.
Data exclusions	Two mice in the SIHUMI+Bw group died on day 40 (considering the total length of the experiment); therefore, any parameters quantified after this date did not include these two mice.
Replication	The animal experiment was repeated twice.
Randomization	Allocation of the germ-free mice was random
Blinding	The bacterial inoculum administered to the mice was blinded to the individuals handling the mice

Reporting for specific materials, systems and methods

We require information from authors about some types of materials, experimental systems and methods used in many studies. Here, indicate whether each material, system or method listed is relevant to your study. If you are not sure if a list item applies to your research, read the appropriate section before selecting a response.

Materials & experimental systems

n/a	Involved in the study
☐	☒ Antibodies
☒	☐ Eukaryotic cell lines
☒	☐ Palaeontology and archaeology
☐	☒ Animals and other organisms
☒	☐ Clinical data
☒	☐ Dual use research of concern
☒	☐ Plants

Methods

n/a	Involved in the study
☒	☐ ChIP-seq
☐	☒ Flow cytometry
☒	☐ MRI-based neuroimaging

Antibodies

Antibodies used	CD45-APC-Cy7 (BD), CD11b-PE (BD) and F4/80-FITC (Myltenyi) antibodies
Validation	All antibodies used are commercially available and validated for flow cytometry in mouse tissue by the manufacturers. CD45-APC-Cy7 (BD, Cat# 557659, dilution 1:200), CD11b-PE (BD, Cat# 553311, dilution 1:200), and F4/80-FITC (Miltenyi, Cat# 130-110-443, dilution 1:100), were used at the dilutions recommended by the manufacturers consistent with Blokker et al., Hepatology 2019 (10.1002/hep.30275). Additional validation data for these antibodies are available on the manufacturers' websites.

Animals and other research organisms

Policy information about studies involving animals; ARRIVE guidelines recommended for reporting animal research, and Sex and Gender in Research

Laboratory animals	C57BL/6 mice, aged 5-6 weeks old. Mice were obtained from an in-house colony at the University of East Anglia. All mice were housed in an experimental isolator at the Disease Modelling Unit, University or East Anglia and maintained under a 12 h light/dark cycle and received autoclaved water and an RM3 (Autoclavable) (GF) diet (Special Diets Services). The high-fat diet comprised 19.5% milk fat (45.2% kcal fat, 41% kcal carbohydrate, 3% SBO, TK VM, blue, TD.200269, Envigo, US) and the increasing daily proportions were 0%, 20%, 40%, 50%, 80%, 100% w/w HF diet to standard chow.
Wild animals	N/a
Reporting on sex	All animals were male. Only male mice were used in this study to minimize potential variability due to hormonal fluctuations, thereby ensuring greater consistency in physiological responses and microbial composition across experimental groups. Future studies should include female mice to assess sex-specific responses.
Field-collected samples	N/A
Ethics oversight	Germ-free animal experiments were done under the project license NB70/8929. This animal experiment was conducted in accordance with the Home Office Animals (Scientific Procedures) Act 1986.

Note that full information on the approval of the study protocol must also be provided in the manuscript.

Plants

Seed stocks	N/A
Novel plant genotypes	N/A
Authentication	N/A

Flow Cytometry

Plots

Confirm that:

- [x] The axis labels state the marker and fluorochrome used (e.g. CD4-FITC).
- [x] The axis scales are clearly visible. Include numbers along axes only for bottom left plot of group (a 'group' is an analysis of identical markers).
- [ ] All plots are contour plots with outliers or pseudocolor plots.
- [ ] A numerical value for number of cells or percentage (with statistics) is provided.

Methodology

Sample preparation	For liver macrophages, Isolated macrophages were stained with CD45-APC-Cy7 (BD), CD11b-PE (BD) and F4/80-FITC (Myltenyi) antibodies.<br>For cytokines from serum, serum was processed using the Mouse Inflammation Panel 13-plex (740446, BioLegend, UK) according to the manufacturer’s instructions.<br>For bacterial quantification:<br>For bacterial cell quantification, 550 µL 1 X PBS was added to the same stool pellets used for 1H-NMR quantification. Samples were vortexed, diluted further 1:10 with 1X PBS, and filtered through a nylon cell strainer with a 70 µm pore size (Corning 431751, Fisher Scientific, UK). An aliquot of 1 µL was used for dilutions of 1:1600, 1:3200, and 1:6400. To stain the bacterial cells, 200 µL of dilutions were mixed with 10 µL 100 X SYBRTM Green I nucleic acid gel stain (S7563, ThermoFisher Scientific, UK) and incubated in darkness for 30 min at room temperature.
Instrument	BD LSR-Fortessa for liver macrophages and inflammatory cytokines; Guava easyCyteTM HT flow cytometer (Luminex) for bacterial counts
Software	FlowJo software (v.10.8.1); InCyte (v.3.4)
Cell population abundance	No fractions were sorted
Gating strategy	For gating, non-stained samples were used to identify the background noise. For inflammatory cytokines, gating was performed according to the manufacturer's instructions.

- [x] Tick this box to confirm that a figure exemplifying the gating strategy is provided in the Supplementary Information.
